# People with autism perceive drastic illusory changes for repeated verbal stimuli

**DOI:** 10.1038/s41598-019-52329-9

**Published:** 2019-11-01

**Authors:** Chihiro Itoi, Nobumasa Kato, Makio Kashino

**Affiliations:** 1 Department of Psychology, Faculty of Letters, Chuo University, 742-1 Higashinakano, Hachioji, Tokyo 192-0393 Japan; 2 Medical Institute of Developmental Disabilities Research, Showa University, Kitakarasuyama 6-11-11, Setagaya, Tokyo 157-8577 Japan; 3 NTT Communication Science Laboratories, 3-1 Morinosato Wakamiya, Atsugi, Kanagawa 243-0198 Japan

**Keywords:** Perception, Autism spectrum disorders

## Abstract

A core symptom of autism spectrum disorder (ASD) is restricted and repetitive behavior, characterized partly by insistence on sameness and excessively focused interest. This behavior has often been interpreted as a manifestation of anxiety and fear triggered by resistance to change. The implicit assumption underlying this interpretation is that perception per se (such as the judgment of sameness and changes in sensory stimuli) is not different between ASD and typically developed (TD) individuals, but that only the emotional response to the same amount of perceived change is. However, few studies have examined how individuals with ASD actually perceive a repeated presentation of the same sensory stimulus. To explore this issue, we conducted a listening test to compare perception of a repeated sound pattern, namely a spoken word, between ASD and TD groups. Prolonged listening to a repeated word without a pause may induce perceptual changes, which is known as the verbal transformation effect. We discovered that individuals with ASD tend to perceive more drastic changes or differences for the same repeated auditory pattern. This suggests that such variable perception incites individuals with ASD to persist for sameness.

## Introduction

Autism spectrum disorder (ASD) is a neurodevelopmental disorder characterized by communication difficulty and restricted and repetitive behavior^1^. The latter is characterized partly by insistence on sameness (e.g., distress at apparently small changes, such as in the packaging of a favorite food) and resistance to change (e.g., repetitively arranging toys in the same way).

This behavior has often been interpreted as a manifestation of anxiety and fear triggered by resistance to change^1,2^. The implicit assumption underlying this interpretation is that perception per se (such as the judgment of sameness and changes in sensory stimuli) is not different between ASD and typically developed (TD) individuals, but that only the emotional response to the same amount of perceived change is.

An alternative hypothesis is that the perception per se of repetitive stimuli is different between ASD and TD individuals. Nearly 90% of individuals with ASD experience atypical sensory processing^3–6^. However, few studies have examined how individuals with ASD actually perceive a repeated presentation of the same sensory stimulus.

To gain insight into this issue, we performed an experiment using an auditory illusion to compare the perception of a repeated sound pattern, namely a spoken word, between ASD and TD groups. Prolonged listening to a repeated word without a pause may induce perceptual changes, which is known as the verbal transformation (VT) effect^7^. For example, the repetitive “tress” may be transformed into a variety of verbal forms, such as “dress”, “stress”, “drest”, or even “Esther”^7–9^. Previous studies have claimed that the mechanisms inducing illusory changes while listening to repeated words might, under conditions of normal speech discourse, lead to greater accuracy of perception^10^. If language skills are reflected in the perception of VTs, individuals with ASD, who often exhibit difficulties in speech perception^11,12^, would also be expected exhibit atypical perception in VT.

Moreover, recent investigations have demonstrated reduced adaptation^13–15^, a lack of normalization^16^, and violation of Weber’s law^17^ in individuals with ASD. These findings suggest the possibility that individuals with ASD insufficiently calibrate an incoming input to its immediate context. Hence, individuals with ASD would expected to show atypical perception in VT (e.g., report fewer forms or a larger variety of them).

Here we compared the perception of VT in 32 adults diagnosed with ASD and 43 neuro-typical controls, matched for age and IQ (Table 1). For five minutes, participants diotically listened through headphones to the word “banana” spoken repeatedly by a female Japanese native speaker, and reported verbally what they perceived whenever they experienced a VT. We recorded the participants’ reports with a voice recorder and transcribed them, and analyzed the quantitative and qualitative nature of the VTs.

**Table 1:** Participant details.

	TD		ASD		*t* test
	Mean	SD	Mean	SD	
Age	28.28	4.23	28.84	3.98	*p* = 0.56
Full Scale IQ	105.09	13.59	108.81	13.71	*p* = 0.25
Verbal IQ	107.51	14.11	114.22	14.39	*p* = 0.05
Performance IQ	101.72	14.47	99.22	16.10	*p* = 0.49
AQ Total	18.63	6.72	34.59	5.89	*p* < 0.01
Social skill	3.58	2.60	7.59	2.06	*p* < 0.01
Attention switching	4.42	1.95	5.66	2.21	*p* < 0.01
Attention to detail	4.05	1.96	7.59	1.79	*p* < 0.01
Communication	2.91	2.26	6.31	1.82	*p* < 0.01
Imagination	3.67	1.87	8.97	2.67	*p* < 0.01

## Results

**Figure 1:**
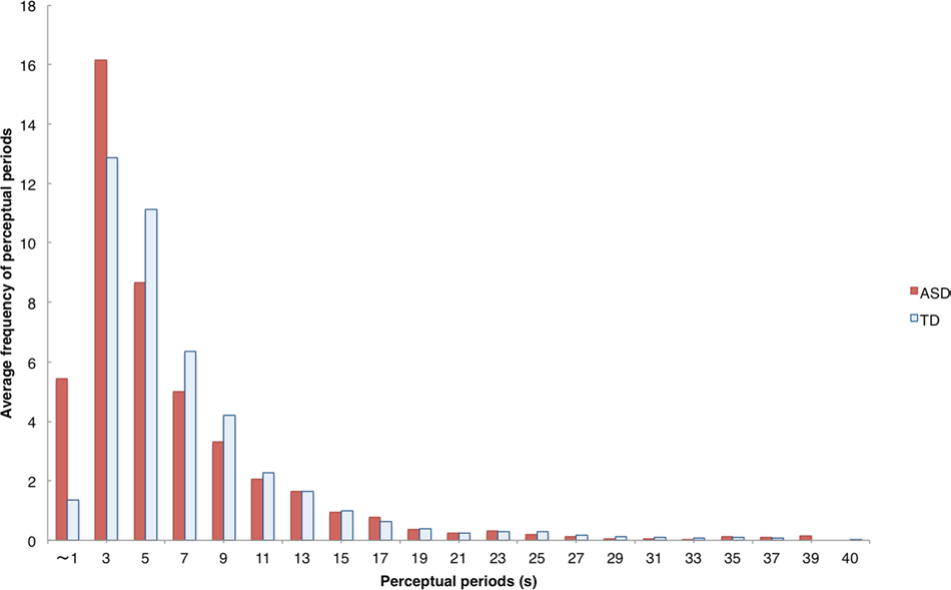
Frequency of perceptual periods per form in TD and ASD groups (for details, see text). Perceptual periods of 40 s or more were excluded from the figure.

First, we analyzed the transcriptions to determine the numbers of transformations and different forms. For example, the forms reported by one TD participant during the five-minute period were “banana… banaN… panaN… panana… panaN… banaN” (“N” is a special mora in the Japanese phonological system), so in this case, the number of transformations was counted as six and the number of forms as four. There was no significant difference in the number of transformations between the TD group (mean = 43.33, SD = 25.98) and ASD group (mean = 45.44, SD = 36.69) groups: *t* = 0.29, *p* = 0.77, *d* = 0.07. The average values of perceptual times per form were 6.44 s (SD = 13.41) in the ASD group and 6.82 s (SD = 12.34) in the TD group. The distribution diagram of perceptual periods per form between individuals with ASD and TD individuals showed that individuals with ASD reported forms for a very short time (within three seconds) (Fig. 1). This result suggests that the reporting criteria of individuals with ASD could differ from those of TDs.

**Figure 2:**
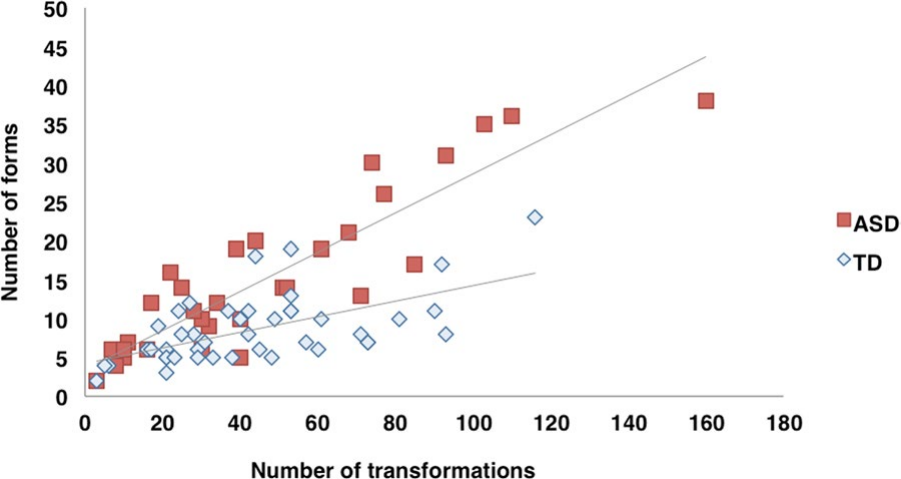
Distribution of the number of forms vs number of transitions in TD and ASD groups (for details, see text).

The number of forms was significantly larger in the ASD group (mean = 14.88, SD = 10.22) than in the TD group (mean = 8.58, SD = 4.39): *t* = 3.26, *p* = 0.002, *d* = 0.85. The distribution of individual differences (the number of transformations vs the number of forms) is shown in Fig. 2. The distribution is not completely separated between ASD and TD groups; however, the slopes of the approximate straight lines are different between the groups. The participants who reported a large number of transformations and forms were individuals with ASD.

**Figure 3:**
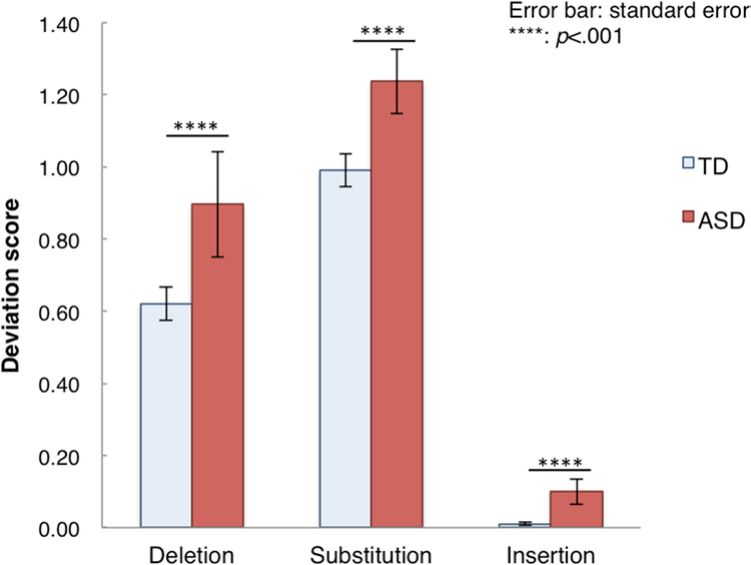
Deviation scores (deletion, substitution, insertion) in TD and ASD groups (for details, see text).

Moreover, we observed that participants with ASD generally reported forms that more drastically deviated from the original form (in this case, “banana”). One ASD participant, for instance, reported “aru… danana… haiNnaino… hairando… heNnano …panama”, and another one reported “banana… peNdaNto… banama… tadano… peNdaNtu… pedanu”. To analyze this observation quantitatively, we introduced a “deviation score”. First, phonemic transformations in the reported forms were categorized into three types of deviation: deletion (e.g., “b” is deleted in the transformation from “banana” to “anana”), substitution (e.g., “b” is displaced in the transformation from “banana” to “panana”), and insertion (e.g., “a” is inserted in the transformation from “banana” to “bananaa”). Second, the number of phonemic transformations in a reported form was counted separately in each category for each participant. For example, when “banana” was transformed into “panaN”, “deletion” was counted as one, “substitution” as one, and “insertion” as zero (Table 2). Then, the deviation score in each category was determined from the sum of the counts in the category for all transformations divided by the total number of transformations for each participant. A two-way mixed ANOVA [group (TD, ASD) × deviation scores (deletion, substitution, insertion)] revealed a significant group difference (*F*(1, 73) = 19.19*, p* < 0.001, partial η^2^ = 0.21), a significant difference in deviation scores (*F*(2, 72) = 635.22*, p* < 0.001, partial η^2^ = 0.95), and no significant interaction (*F*(2, 146) = 0.92*, p* = 0.40) (Fig. 3). This analysis confirmed that participants with ASD generally reported forms that more drastically deviated from the original form.

**Table 2:** Examples of scores of calculated deviation (deletion, substitution, insertion) from the original form. (A) A TD participant; (B) An ASD participant (“N” is a special mora in the Japanese phonological system).

	Deletion	Substitution	Insertion
**(A)**			
banana	0	0	0
banaN	1	1	0
panaN	1	2	0
panana	0	1	0
panaN	1	2	0
banaN	1	1	0
Sum of counts for all word transformations	4	7	0
Total number of word transformations	6	6	6
Sum of counts for all word transformations /total number of word transformations	4/6	7/6	0/6
Deviation scores	0.67	1.17	0
**(B)**			
banana	0	0	0
peNdaNto	0	5	2
banama	0	1	0
tadano	0	3	0
peNdaNtu	0	5	2
pedanu	0	4	0
Sum of counts for all word transformations	0	18	4
Total number of word transformations	6	6	6
Sum of counts for all word transformations /total number of word transformations	0/6	18/6	4/6
Deviation scores	0	3.0	0.67

To assess the relationship between language skills (VIQ in WAIS) and the results of the VT, we conducted a correlation analysis between VIQ and the number of transformations, number of forms and deviation scores. VIQ did not correlate with the results of the VT (*r* = 0.09, *p* = 0.45 for the number of transformations; *r* = 0.15, *p* = 0.19 for the number of forms; *r* = 0.06, *p* = 0.64 for deviation scores).

**Figure 4:**
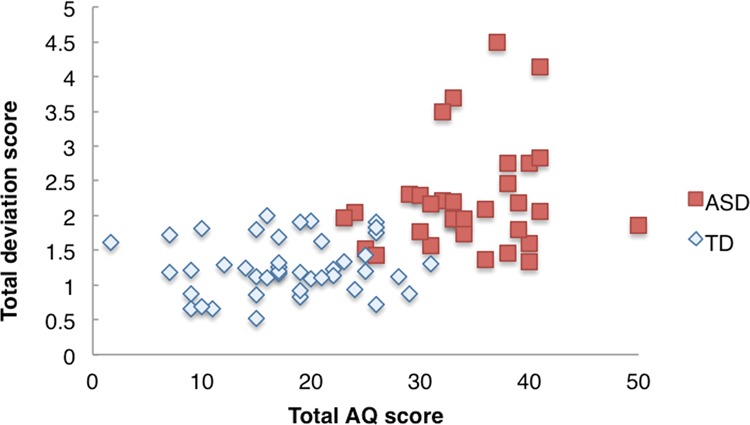
Distribution of the total AQ score and deviation score in TD and ASD groups (for details, see text).

To assess the relationship between self-reported autistic traits (Autism Quotient: AQ)^18^ and the results of the VT, we conducted a correlation analysis between the AQ scores and number of transformation, number of forms and the deviation scores for all participants. There was a significant correlation between the total AQ scores and the deviation scores (*r*_p_ = 0.58, *p* < 0.001). As the components of the AQ scores, the deviation scores did not correlate with attention to detail (*r*_p_ = 0.19, *p* = 0.11), but other components correlated with the deviation scores (*r*_p_ = 0.50, *p* < 0.001 for social skill; *r*_p_ = 0.45, *p* < 0.001 attention switching; *r*_p_ = 0.54, *p* < 0.001 for communication; *r*_p_ = 0.50, *p* < 0.001 for imagination). Figure 4 shows the distribution of the deviation scores and total AQ scores for all participants. There was no correlation between the deviation scores and AQ scores in either group (ASD, TD). The distribution of the deviation scores and AQ scores overlapped between individuals in both groups, though there was a region where they are distributed by only individuals with ASD.

**Figure 5:**
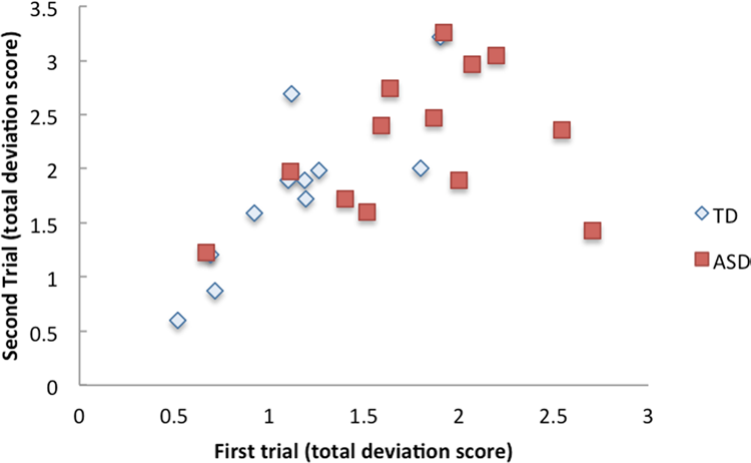
Distribution of the deviation score in TD and ASD groups in first and second trials (for details, see text).

We conducted a VT experiment on another day to investigate whether individuals with ASD have increased intra-individual variability. Thirteen individuals with ASD and 11 TD individuals, all of whom participated in the first experiment, participated in the second VT experiment. A two-way mixed ANOVA was performed, with group (ASD, TD) as a between-subject factor and trial (the sum of deviation scores in the first and second trials) as a within-subject factor. There was no significant difference in trials between ASD and TD groups (*F*(1, 22) = 0.74, *p* = 0.40). There was a significant main effect of group (*F*(1,22 = 6.49, *p* = 0.02, partial η^2^ = 0.23) and trial (*F*(1, 22) = 20.57, *p* < 0.001, partial η^2^ = 0.48). The correlation analysis between trials and the sum of deviation scores revealed a high correlational relationship in the TD group (*r*_p_ = 0.83, *p* = 0.002) but no correlational relationship in the ASD group (*r*_p_ = 0.35, *p* = 0.24). Figure 5 shows the distribution of the deviation scores in trials for the ASD and TD groups. This analysis confirmed consistency in the degree of deviation across trials for TD individuals. On the other hand, individuals with ASD reported more variable forms across trials.

## Discussion

This study revealed that individuals with ASD tend to report more drastic changes or differences for an unchanging, repeated auditory pattern. This finding provides a fresh view of the apparent insistence on sameness that individuals with ASD often show. In the conventional view, it has been implicitly assumed that perception per se is not different between individuals with ASD and those with TD and that a physically unchanging stimulus is perceived as unchanging by both groups. Apparently, these assumptions are not valid. As we have shown here, individuals with ASD experienced more drastic perceptual changes for a repeated stimulus than TD individuals did. In other words, the insistence on sameness in individuals with ASD is at least partly due to their uniqueness in perception, not solely due to their uniqueness in preference. For individuals with ASD, a physically unchanging, repeated pattern may be evaluated as producing the “right amount of” perceptual change. This may explain why individuals with ASD show apparent insistence on sameness.

It is known that individuals with ASD have a tendency to pay more attention to finer details than TD individuals do. Therefore, they may notice subtle differences in sensory information due to internal noise or neural adaptation. However, if this were the cause of illusory changes that the individuals with ASD experienced, the perceived forms would not have been very different from the original form.

Previous studies have reported atypical perceptual processing in ASD. It has been shown that individuals with ASD are less susceptible to geometric illusions^19–21^ and exhibit reduced adaptation^13–15^, resulting in veridical perception^22^. A natural expectation from these findings is that individuals with ASD would perceive a smaller variety of forms or less deviated forms in VT for repeated verbal stimuli than TD individuals would. However, in the present study, not only did individuals with ASD report a larger variety of forms, but their reported forms contained drastic perceptual changes as well. A recent study suggested that susceptibility to perceptual illusions is not an all or nothing phenomenon and may depend on the specific nature of the perceptual process involved^23^. Indeed, individuals with ASD exhibit clear susceptibility to perceptual illusions, at least in some cases^17,24^. The results of the present study are in line with these studies.

Then, what is the cause of the drastic illusory changes in individuals with ASD? It should be noted here that VT is determined not only by bottom-up sensory information but also by top-down predictive information, such as articulatory constraints and semantic knowledge^9,10,25–27^. This is supported by the findings that VT involves the interaction of widely distributed networks in the brain, including the auditory cortex, inferior frontal cortex (Broca’s area), anterior cingulate cortex, and caudate^27^. A plausible explanation would therefore be that the effect of top-down prediction in VT is stronger in individuals with ASD than in TD individuals.

It has often been pointed out that individuals with ASD have a weaker top-down effect. This has been interpreted in a predictive coding framework as hypo-prior^22^ or high weighting for prediction error^28^. The present findings suggest an apparently opposite direction. This apparent inconsistency would be resolved by assuming inappropriate gain control for the evaluation of prediction error. If the gain is too low, the weighting to prediction error gets small, resulting in an increased effect of top-down prediction. If it is too high, on the other hand, the weighting to prediction error gets large, resulting in a decreased effect of top-down prediction. So far, only the latter case has been considered. Here, we have shown that both cases may occur in the perception of individuals with ASD.

The gain control could be implemented neurally by the balance between excitation and inhibition (E/I balance)^5,16^. It has been shown that the E/I balance tends to be inappropriate in individuals with ASD^29^. Moreover, fMRI studies have demonstrated that neural responses to repetitive stimuli in individuals with ASD have greater intra-individual variability compared to TD individuals^30,31^. This could be one of the causes of the atypical perception to repeated stimuli, which may contribute to the insistence on sameness that individuals with ASD often exhibit. The results of our study suggest there may be larger intra-individual variability in individuals with ASD than in TD individuals, though the number of samples may not be large enough to perform a correlation analysis of intra-individual perceptual variability. To more fully reveal the variability between trials in individuals with ASD, further research is needed with a larger the number of data samples is needed.

One may argue that poor verbal skills are reflected in the deviated forms from individuals with ASD. However, this was not the case. We used VIQ scores in WAIS to assess their language skills and found that individuals with ASD exhibited higher verbal ability than TD individuals. Moreover, there was no relationship between language skills and the VT results, though there was a significant correlation between deviation scores and AQ scores. These results suggest that the drastically deviated changes in VT in individuals with ASD are related to ASD traits rather than language skills.

The present findings could not only change how we view the traits of people with ASD, but also provide a clue to revealing the functional mechanisms underlying their unique perceptual experiences. Further research is needed to explore this possibility.

## Methods

### Participants

Participants were 32 high-functioning adults with ASD (four females) and 43 TD adults (nine females). Informed consent was obtained from all participants. They were matched in age (mean ± SD: ASD group, 28.8 ± 4.0; TD group, 28.3 ± 4.3) and IQ (FIQ: ASD group, 108.8 ± 13.7; TD group, 107.5 ± 14.1). As a screening tool, we used the Japanese version of the Autism-Spectrum Quotient (AQ) test^18^. None of the TD adults displayed clinically significant levels of autistic symptomatology, as indexed by the AQ. The control participants had no history of psychiatric illness or neurological disorders.

ASD participants were recruited from outpatient units of Karasuyama Hospital, Tokyo, Japan. The diagnosis of ASD was based on a consensus reached by three experienced psychiatrists according to the criteria of the Diagnostic and Statistical Manual of Mental Disorders (DSM-5), Fifth Edition.

All procedures were conducted in accordance with the Declaration of Helsinki and approved by the Ethics Committee of the NTT Communication Science Laboratories and Showa University Karasuyama Hospital. The participants were paid for their time.

### Stimuli and procedure

Stimuli for the 300-s sessions were 884 repetitions of the word “banana” spoken by a female native speaker of Japanese. Participants listened to the word without gaps at a comfortable level diotically through headphones (Sennheiser HDA200). While listening to the stimulus, they were instructed to close their eyes and report the form when a perceptual transition from one verbal form to another occurred. Furthermore, if the perceptual form was reversed from a new form to the original form, participants were asked to report the original form. They were asked not to repeat the stimulus word in their mind. The responses from participants were recorded with a voice recorder (ICD-UX543F, SONY) and transcribed by the experimenter.

### The number of transformations and different forms

All forms reported by the participants during the 300-s sessions were counted as the number of transformations. Different kinds of forms reported by participants during the 300-s sessions were counted as the number of different forms. For example, for participants who reported “ banaN…banana…banaN…”, the number of transformations was counted as three and the number of different forms as two.
